# Transformation and Characterization of Δ12-Fatty Acid Acetylenase and Δ12-Oleate Desaturase Potentially Involved in the Polyacetylene Biosynthetic Pathway from *Bidens pilosa*

**DOI:** 10.3390/plants9111483

**Published:** 2020-11-03

**Authors:** Po-Yen Chen, Mi-Jou Hsieh, Yung-Ting Tsai, Hsiao-Hang Chung, Lie-Fen Shyur, Cheng-Han Hsieh, Kin-Ying To

**Affiliations:** 1 Agricultural Biotechnology Research Center, Academia Sinica, Taipei 115, Taiwan; Chen-Po-Yen@hotmail.com (P.-Y.C.); lapinnemi@gmail.com (M.-J.H.); soloduo@gate.sinica.edu.tw (Y.-T.T.); lfshyur@ccvax.sinica.edu.tw (L.-F.S.); james0502@gate.sinica.edu.tw (C.-H.H.); 2 Department of Horticulture, National Ilan University, Yilan 260, Taiwan; hhchung@niu.edu.tw; 3 PhD Program in Translational Medicine, College of Medicine, Kaohsiung Medical University, Kaohsiung 807, Taiwan

**Keywords:** polyacetylenes, *Bidens pilosa*, acetylenase, desaturase, *Agrobacterium*-mediated transformation, *FAD2*, medicinal herb, transgenic plants

## Abstract

*Bidens pilosa* is commonly used as an herbal tea component or traditional medicine for treating several diseases, including diabetes. Polyacetylenes have two or more carbon–carbon triple bonds or alkynyl functional groups and are mainly derived from fatty acid and polyketide precursors. Here, we report the cloning of full-length cDNAs that encode Δ12-fatty acid acetylenase (designated BPFAA) and Δ12-oleate desaturase (designated BPOD) from *B. pilosa*, which we predicted to play a role in the polyacetylene biosynthetic pathway. Subsequently, expression vectors carrying BPFAA or BPOD were constructed and transformed into *B. pilosa* via the *Agrobacterium*-mediated method. Genomic PCR analysis confirmed the presence of transgenes and selection marker genes in the obtained transgenic lines. The copy numbers of transgenes in transgenic lines were determined by Southern blot analysis. Furthermore, 4–5 *FAA* genes and 2–3 *OD* genes were detected in wild-type (WT) plants. Quantitative real time-PCR revealed that some transgenic lines had higher expression levels than WT. Western blot analysis revealed OD protein expression in the selected transformants. High-performance liquid chromatography profiling was used to analyze the seven index polyacetylenic compounds, and fluctuation patterns were found.

## 1. Introduction

*Bidens pilosa* L., which belongs to the Asteraceae family, is an erect annual plant. It is native to South America and is now widely distributed in subtropical and tropical regions of the world. *B. pilosa* is commonly used as an herbal tea component or as traditional medicine in Latin America, Africa, and Asian countries for treating various disorders, such as inflammation, stomach illnesses, malaria, liver disorders, enteritis, dysentery, diabetes, and hypertension [1,2,3]. In Taiwan, three variants (namely, *radiata, pilosa,* and *minor*) of *B. pilosa* are often used as a folk medicine for curing diabetes. However, better anti-diabetic properties were observed in the plant extract from the variant *radiata* as compared with the other two variants [4]. To date, around 200 secondary metabolites have been identified from *B. pilosa*, including polyacetylenes, flavonoids, phenylpropanoids, and terpenes; polyacetylenes and flavonoids are two predominant classes of metabolites [5]. Among these metabolites, bioactive polyacetylenic compounds, such as glycosides or aglycones, with functions against type I diabetes or angiogenesis have been identified [6,7,8,9].

Polyacetylenes have two or more carbon–carbon triple bonds or alkynyl functional groups and are mainly derived from fatty acid and polyketide precursors, and polyacetylenic compounds possess antibacterial, antifungal, or antitumor properties [10,11]. More than 2000 polyacetylenes have been identified, and more than 1100 compounds with diverse acetylenic structures have been found in the plant family Asteraceae [12]. The crepenynate pathway has been suggested to be a major route for polyacetylene biosynthesis and has been investigated in fungi and plants over the past several decades [11,12]. Several genes related to this pathway, such as the desaturases and acetylenases, have been cloned and characterized. For instance, the soluble stearoyl-ACP desaturase (SAD) can convert a single bond to a double bond at the C-9 position of stearic acid (C18:0) to form oleic acid (C18:1), and microsomal oleate Δ12-desaturase (named FAD2 in plants) catalyzes desaturation at the C-12 position of oleic acid to form linoleic acid (C18:2). The first plant *FAD2* gene was cloned from *Arabidopsis thaliana* [13]. Subsequently, orthologous DNA sequences were identified and characterized in a range of plant species. Only a single *FAD2* gene has been detected in *Arabidopsis* [13], whereas multiple members of the *FAD2* gene family (such as desaturases, hydrogenases, epoxygenases, acetylenases, and conjugases) with diverse functional activities in fatty acid modification have been reported in most plant species [14,15]. For example, two *FAD2* genes have been reported in olive (*Olea europaea*) [16] and tomato [17], three in soybean [18], sunflower [19] and *Medicago truncatula* [20], four in cotton [21,22] and oilseed (*Brassica napus*) [23], and six in peanut (*Arachis hypogaea*) [24]. Remarkably, eleven and twenty-six members of the *FAD2* gene family were identified and characterized in safflower [14] and desert shrub *Artemisia sphaerocephala* [15], respectively. The other key enzyme, Δ12 acetylenase, catalyzes the conversion of a double bond to a triple bond, which converts linoleic acid to crepenynic acid [25]. These studies confirmed that desaturations are a major reaction for alkyne bond formation. Further modification of the acetylenic backbones, such as chain elongation, oxidative cleavage processes, and hydroxylation or dehydrogenation, can produce numerous polyacetylenic metabolites that vary in chain length or fine structure [12,26]. The *FAD2* gene family is the key step in the accumulation of polyunsaturated fatty acids; moreover, it plays an essential role in the membrane integrity of cell membranes and is often induced in response to various biotic and abiotic stresses, such as extreme temperatures, high salinity, and pathogen attack [14,17]. Although recent studies have shown that the formation of the alkyne bond of polyacetylenes involves catalysis by desaturases and acetylenases, the complete picture of the biosynthesis pathway of the polyacetylene class is still obscure. The broad bioactivities of polyacetylenic natural products, as well as their considerable benefit to human and animal health, reflect the importance of these polyacetylenes; it is therefore important to explore their biosynthetic route, especially in the ethnopharmacologically important medicinal plant *B. pilosa*.

In this study, we report the first cloning of full-length cDNAs that encode Δ12-fatty acid acetylenase and Δ12-oleate desaturase, which we predicted to be key genes in the polyacetylene biosynthesis pathway, from the medicinal plant *B. pilosa* var. *radiata*. Subsequently, *Agrobacterium*-mediated transformation was carried out in *B. pilosa* var. *radiata*. Molecular characterization was performed among these transgenic plants.

## 2. Results

### 2.1. Cloning and Sequence Analysis of Putative Δ12-Oleate Desaturase and Δ12-Fatty Acid Acetylenase Genes from Bidens pilosa var. radiata

**Figure 1:**
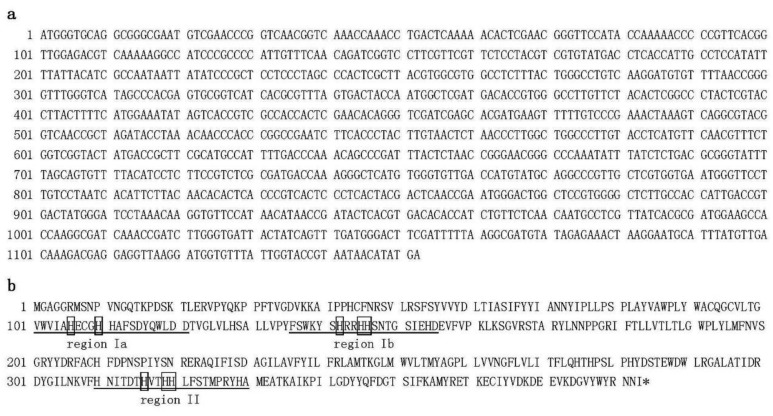
Nucleotide sequence (**a**) and deduced amino acid sequence (**b**) that encode Δ12-oleate desaturase (BPOD; accession number MF318524) from *Bidens pilosa* var. *radiata*. Eight conserved histidine residues are indicated in boxes. Three regions (region Ia, region Ib, region II) of catalytic sites are underlined.

Green leaves from *B. pilosa* var. *radiata* were harvested, total RNA was isolated, and SMART RACE cDNA amplification was carried out to clone the full-length cDNAs that encode Δ12-oleate desaturase (designated BPOD) and Δ12-fatty acid acetylenase (designated BPFAA) in the polyacetylene biosynthetic pathway. The BPOD cDNA (Figure 1) is 1152 bp in length and contains a reading frame of 383 amino acids (aa) with a predicted pI of 8.55 and predicted molecular weight (MW) of 44122 Da. Our clone is the same length as the published Δ12 oleate desaturase (FAD2-2) mRNA from sunflower (*Helianthus annuus*) (GenBank accession no. AF251843) [19]. Nucleotide sequence alignment and protein sequence alignment between sunflower FAD2-2 and our BPOD clone revealed 87% and 93% sequence identity, respectively. In addition, the deduced protein sequence from our BPOD cDNA clone also showed homology to other desaturases. Furthermore, three regions of conserved histidine cluster motifs that contain eight histidine residues—HXXXH, HXX(X)H, and HXXHH [27]—were also found in our BPOD clone (Figure 1b). These histidine residues are catalytic sites and proposed to be the ligands for the iron atoms in stearoyl CoA desaturase, a membrane-associated enzyme [27]. The predicted function of BPOD is the conversion of oleic acid to linoleic acid.

**Figure 2:**
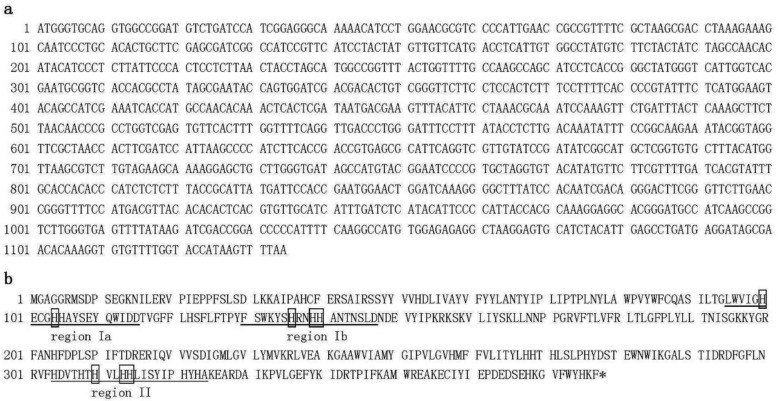
Nucleotide sequence (**a**) and deduced amino acid sequence (**b**) that encode Δ12-fatty acid acetylenase (BPFAA; accession number MF318525) from *Bidens pilosa* var. *radiata*. The eight conserved histidine residues are indicated by the boxes. Three regions (region Ia, region Ib, region II) of the catalytic sites are underlined.

The other clone, BPFAA (Figure 2), is 1134 bp in length and contains a reading frame of 377 aa with a predicted pI of 8.19 and predicted MW of 43,935 Da. This clone is the same length as the published Δ12 fatty acid acetylenase (*FAA*) from sunflower (*H. annuus*) (GenBank accession no. AY166773; [28]). Nucleotide sequence alignment and protein sequence alignment of sunflower *FAA* and our BPFAA clone revealed 85% and 92% sequence identity, respectively. FAA is recognized as a triple-bond-forming enzyme, catalyzing the conversion of linoleate into crepenynate through acetylenation at the Δ12 position. It has been reported that overexpression of this enzyme in transgenic soybean seeds leads to the accumulation of crepenynic and dehydrocrepenynic acids, two Δ12-acetylenic fatty acids [28]. Protein sequence alignment of our BPOD and BPFAA clones revealed 61% sequence identity; moreover, the eight conserved histidine residues in the three regions were also found in our BPFAA clone (Figure 2b).

These two full-length cDNAs were cloned separately into the Gateway expression vector pK2GWIWG2, resulting in pBPFAA (Supplementary Figure S1a) and pBPOD (Supplementary Figure S1b). These vectors were transformed separately into *A. tumefaciens* strain LBA4404.

### 2.2. Phylogenetic Analysis of Δ12-Oleate Desaturase and Δ12-Fatty Acid Acetylenase Genes from Bidens pilosa var. radiata

**Figure 3:**
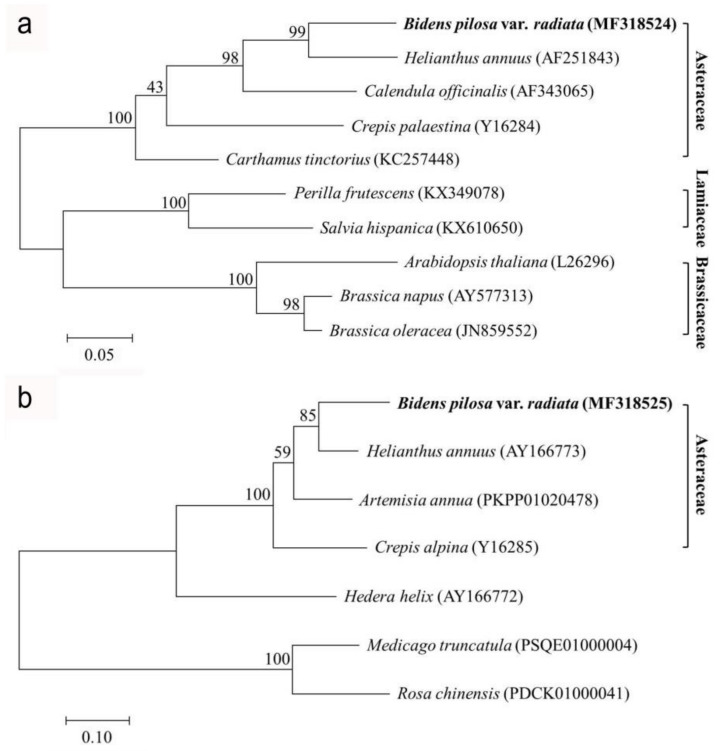
Phylogenetic relationships of (**a**) oleate desaturase (OD) and (**b**) fatty acid acetylenase (FAA) genes based on their complete coding DNA sequences (CDSs). The numbers in brackets are the accession numbers of the OD and FAA sequences. Molecular phylogenetic analyses were conducted using the Maximum Likelihood method based on the Tamura–Nei model [29]. Bootstrap values at the nodes are the percentages of 1000 replicates.

The phylogenetic tree of OD (Figure 3a) with 99% bootstrap replicates indicates that our BPOD is closest to *Helianthus annuus* (GenBank accession no. AF251843) and is grouped with other Asteraceae species. Sequences from three families, Asteraceae, Lamiaceae, and Brassicaceae, clearly form three different clades. Although there are not many complete plant *FAA* genes available in the GenBank database at the moment, we observed that the phylogenetic trees of FAA from all of the Asteraceae species are clustered in one clade, and BPFAA is closest to *H. annuus* (GenBank accession no. AY166773) (Figure 3b).

### 2.3. Confirmation of Putative Transformants

**Figure 4:**
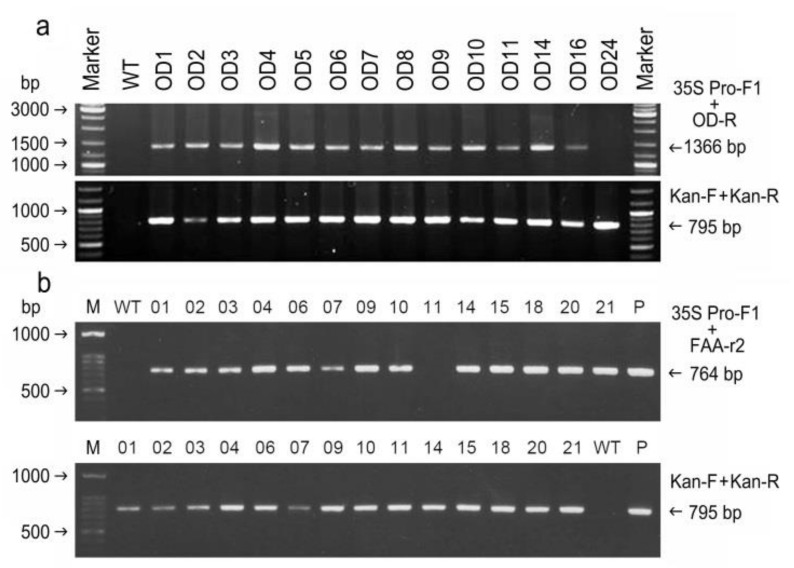
Verification of transgenic OD and FAA plants. (**a**) Genomic PCR analysis of transgenic OD plants. Primers 35 S Pro-F1 and OD-R were designed to detect the DNA fragment (1366 bp) of the CaMV 35 S promotor/OD region, while primers Kan-F and Kan-R were designed to detect the kanamycin resistance gene (*nptII*; 795 bp). (**b**) Genomic PCR analysis of transgenic FAA plants. Primers 35 S Pro-F1 and FAA-r2 were designed to detect the DNA fragment (764 bp) of the CaMV 35 S promotor/FAA region, while primers Kan-F and Kan-R were designed to detect the *nptII* gene (795 bp).

After *Agrobacterium*-mediated transformation and selection, over 10 putative transformants from each transformation vector were obtained and grown in a greenhouse. Typical images of selection and plant regeneration are shown in Supplementary Figure S2. No morphological differences between the WT and all transformants were observed. To verify the transformants, genomic DNA from the leaves of plantlets was isolated, and PCR analysis was carried out. Among the 14 putative OD transgenic plants, a PCR amplicon of 1366 bp, comprising a partial sequence (234 bp) of the cauliflower mosaic virus (CaMV) 35S promoter followed by a partial sequence (1132 bp) of BPOD, was detected in 13 out of the 14 samples that we examined (Figure 4a). No PCR band was detected in the wild-type (WT) sample or the transgenic OD24 line (Figure 4a). However, a unique PCR amplicon of 0.8 kb (*nptII* for the kanamycin selection marker) was detected in all 14 transgenic OD lines but not in the WT (Figure 4a). Similarly, a unique amplicon of 764 bp, comprising a partial sequence (234 bp) of the CaMV 35S promoter and a partial sequence (530 bp) of BPFAA, was detected in 13 out of 14 transgenic FAA lines (Figure 4b). Again, a unique PCR amplicon of 0.8 kb (*nptII*) was detected in all 14 transgenic FAA lines but not in the WT (Figure 4b).

**Figure 5:**
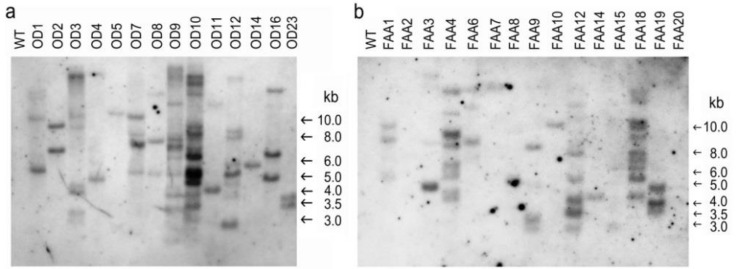
Southern blot analysis. (**a**) Southern blot analysis of transgenic OD plants. (**b**) Southern blot analysis of transgenic FAA plants. Twenty micrograms of DNA was digested with *Eco*RI and probed with the Digoxigenin (DIG)-labeled PCR product of *nptII* (795 bp).

Furthermore, genomic DNA from WT and transgenic plants was digested with *Eco*RI and then probed with the selection marker *nptII* in order to estimate the copy number of the transgene (Figure 5). For the transformation vector pBPOD (Figure 5a) or pBPFAA (Figure 5b), no internal *Eco*RI restriction site in *nptII* was found. As expected, no hybridization band was detected in the WT sample in either Southern blot. For OD transformants, the hybridization bands in all samples were different, indicating that all 14 OD transgenic plants that we obtained in this study were independent integration events (Figure 5a). A single copy of the *nptII* selection marker gene was integrated into the genome of transgenic plants OD5 and OD14; two or more copies of the *nptII* transgene were found in other transgenic plants (Figure 5a). For the FAA transformants, the hybridization bands in all the samples were different, indicating that all 15 FAA transgenic plants were independent integration events (Figure 5b). A single copy of the *nptII* transgene was integrated into the genome of transgenic plants FAA7, FAA10, and FAA14; two or more copies of the *nptII* transgene were found in other transgenic plants (Figure 5b).

**Figure 6:**
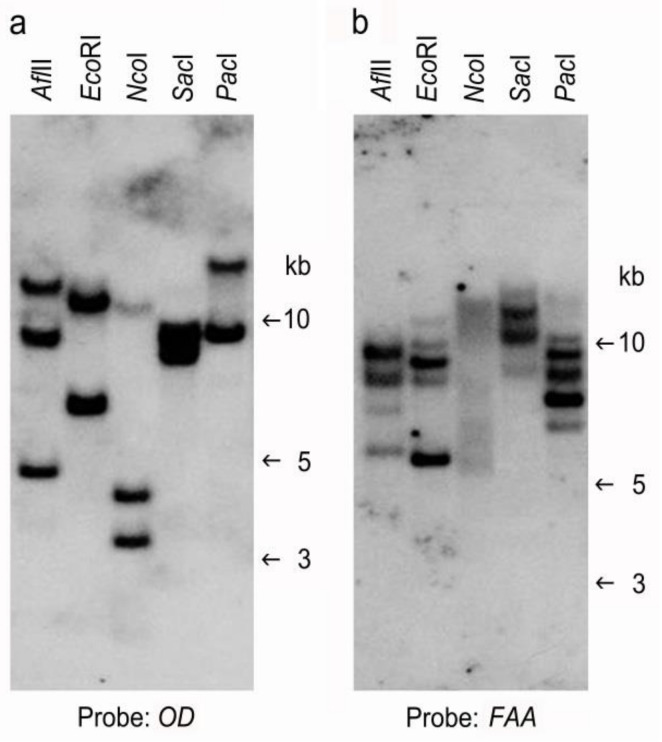
Examination of endogenous FAA and *OD* genes in wild-type *B. pilosa* by Southern blot analysis. Genomic DNA was isolated from wild-type *B. pilosa*, digested with various restriction enzymes as indicated, and probed with full-length *FAA* cDNA (**a**) or full-length *OD* cDNA (**b**).

Meanwhile, to determine the endogenous copies of *FAA* and *OD*, DNA from WT *B. pilosa* was digested with various restriction enzymes and probed with *OD* (Figure 6a) or *FAA* (Figure 6b). No internal restriction sites for *Afl*II, *Eco*RI, *Nco*I, *Sac*I, or *Pac*I were found in the *OD* or *FAA* cDNA sequence. Two to three hybridization bands were detected when the digested DNA sample was probed with *OD* (Figure 6a), suggesting the presence of two to three copies of the *OD* gene in the genome of *B. pilosa*. Four to five hybridization bands were detected when the digested DNA sample was probed with *FAA* (Figure 6b), suggesting the presence of four to five copies of the *FAA* gene in the genome of *B. pilosa*. To examine whether introns were present in our *FAA* and *OD* genes, genomic PCR analysis was performed using genomic DNA from the WT as well as plasmids from pBPFAA and pBPOD as templates. As shown in Supplementary Figure S3, the predicted size of the PCR product (1134 bp) from the pBPFAA plasmid (lane 3) is equal or very similar in size to the PCR product from the WT (lane 2), suggesting that our *FAA* gene may not contain an intron. In addition, the predicted size of the PCR product (1152 bp) from the pBPOD plasmid (lane 6) is equal or very similar in size to the PCR product from the WT (lane 5), suggesting that our *OD* gene may not contain an intron.

### 2.4. mRNA and Protein Expression in Transgenic Plants

**Figure 7:**
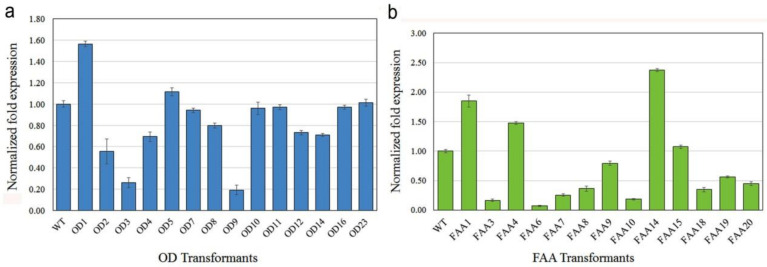
Quantitative real-time PCR analysis in transgenic plants. (**a**) *OD* mRNA expression in leaf tissue of transgenic OD plants. (**b**) *FAA* mRNA expression in leaf tissue of transgenic FAA plants. The results from three or more independent experiments are presented as mean ± S.D. (standard deviation).

The expression of foreign genes in transgenic plants was first analyzed by qRT-PCR analysis (Figure 7). Most OD transgenic plants had similar or lower *OD* mRNA levels as compared with the WT, and around 20% expression level was found in transgenic plants OD3 and OD9 (Figure 7a). Two transgenic plants, OD1 and *OD*5, had higher *OD* mRNA levels as compared with the WT (Figure 7a). For FAA transformants (Figure 7b), only three transgenic plants (FAA1, FAA4, and FAA14) had higher *FAA* mRNA levels as compared with the WT; most FAA transgenic plants had similar or lower *FAA* mRNA levels as compared with the WT. An expression level of around 20% or lower was detected in transgenic plants FAA3, FAA6, and FAA10 (Figure 7b).

**Figure 8:**
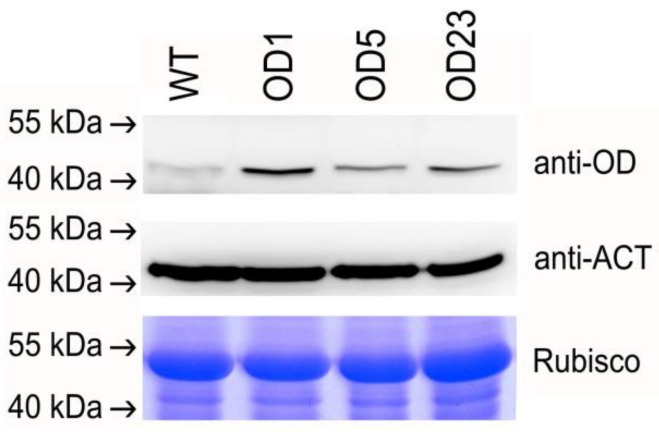
Western blot analysis of selected transgenic OD plants.

To evaluate the presence and accumulation of OD protein in transgenic plants, total protein was extracted from the leaf tissue of selected OD transgenic plants (OD1, OD5, and OD23), which had higher *OD* mRNA expression levels, as revealed by qRT-PCR analysis (Figure 7a), and subjected to Western blot analysis (Figure 8). The protein expression of the *OD* gene in the WT and selected transgenic plants was confirmed by the presence of a 44-kDa band that was specific for the OD protein of *B. pilosa* (Figure 8). Among the transgenic plants, the highest protein level was observed in OD1, and this is consistent with the highest mRNA expression, as revealed by qRT-PCR analysis (Figure 7a). We also tried to examine the FAA protein level in FAA transgenic plants by using the anti-FAA antibody in the western blot analysis; however, the attempt was unsuccessful (data not shown).

### 2.5. Polyacetylenic Compound Profiling in Transgenic Plants

**Figure 9:**
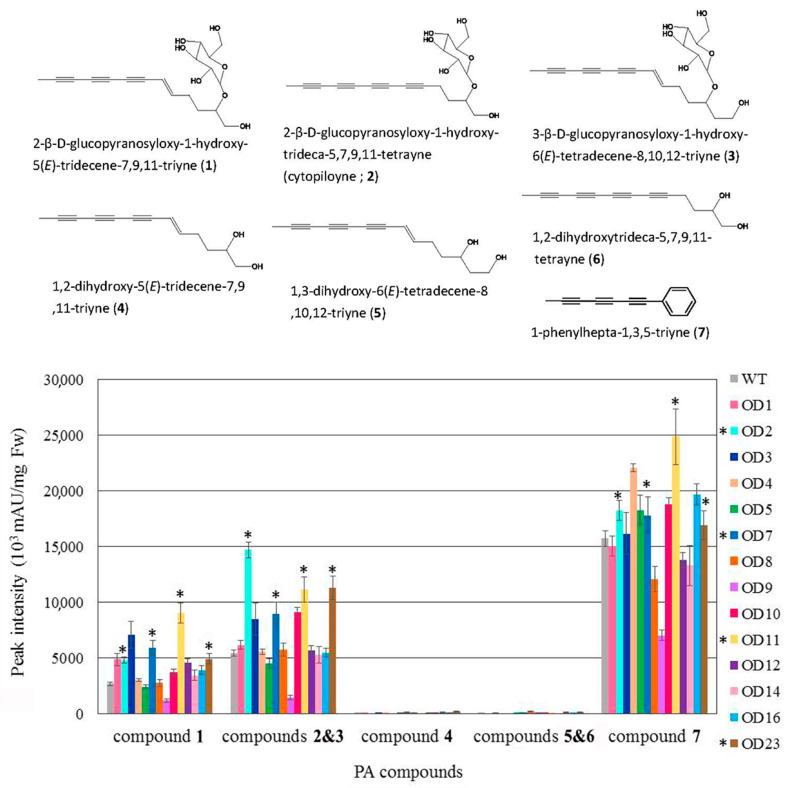
Metabolite profiling in leaf tissue from wild-type and OD transformants of *B. pilosa.* The symbol * indicates higher content of the polyacetyenic (PA) compound as compared with WT. The name and chemical structure of each PA compound are shown at the top of the figure. The results from three or more independent experiments are presented as mean ± S.D.

**Figure 10:**
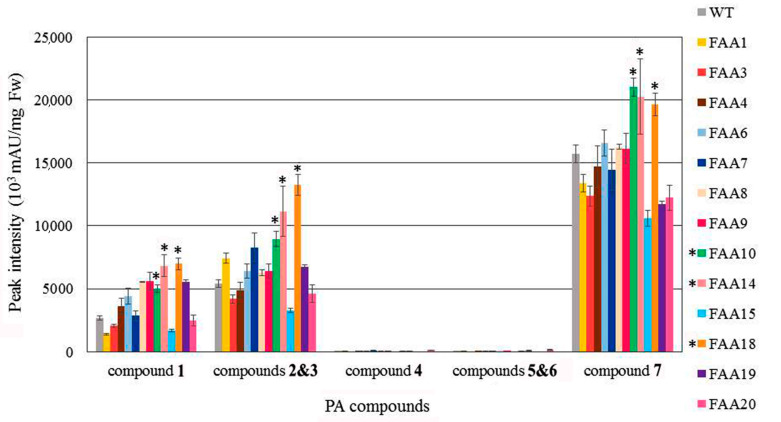
Metabolite profiling in leaf tissue from wild-type and FAA transformants of *B. pilosa.* The symbol * indicates higher content of the polyacetyenic (PA) compound as compared with WT. The name and chemical structure of each PA compound are indicated in Figure 9. The results from three or more independent experiments are presented as mean ± S.D.

Transgenic and WT plants were grown to maturity, and one-month-old leaves were excised for HPLC analysis. As shown in Figure 9, the seven polyacetylenic (PA) compounds were present at higher levels in transgenic plants OD2, OD7, OD11, and OD23 than in the WT. Using the same HPLC approach, the amounts of the seven PA compounds in transgenic plants FAA10, FAA14, and FAA18 were found to be higher than those in the WT (Figure 10). Representative HPLC profiles of the WT and a few transformants are shown in Supplementary Figure S4.

## 3. Discussion

Previously, we established optimal conditions for tissue culture, plant regeneration, and *Agrobacterium*-mediated transformation in *B. pilosa var. radiata* [30]. In this study, expression vectors (Supplementary Figure S1) containing two full-length cDNAs that encode Δ12-oleate desaturase (pBPOD) and Δ12-fatty acid acetylenase (pBPFAA) were transformed independently into *B. pilosa* var. *radiata* according to the protocol detailed in our previous paper [30]. Over 10 transgenic plants were obtained from each transformation vector. Genomic PCR analysis (Figure 4) revealed that all examined FAA and OD transformants, without exception, contained the foreign selection marker gene *nptII* in the transgenic plant genome. Moreover, Southern blot analysis (Figure 5) revealed that all of the examined transformants, without exception, contained at least one copy of *nptII* in the transgenic plant genome, and the T-DNA integration site in each transformant was different. The transformation vector pBPFAA (Supplementary Figure S1a) contains the expression cassettes of *FAA* cDNA and the kanamycin resistance gene *nptII* in the T-DNA region, and the transformation vector pBPOD (Supplementary Figure S1b) contains the expression cassettes of *OD* cDNA and *nptII* in the T-DNA region. Thus, the copy number of *nptII*, which is detected in Figure 5, should be the same as that of the *OD* gene in transgenic OD plants (Figure 5a) or the same as that of the *FAA* gene in transgenic FAA plants (Figure 5b). However, in transgenic plant FAA11, the presence of *nptII* was detected, but the chimeric promoter/FAA expression cassette was not (Figure 4b). Similarly, in transgenic plant OD24, the presence of *nptII* was detected, but the chimeric promoter/OD expression cassette was not (Figure 4a). In brief, incomplete integration of T-DNA into the plant chromosome during the transformation process was found in transgenic plants OD24 and FAA11. Previously, using the transformation vector pCHS, which carries the *Petunia* chalcone synthase (*chs*) and *nptII* genes, we also observed incomplete integration of T-DNA in several plant species, including *B. pilosa* [30], the floricultural plant *Cleome spinosa* [31], and the medicinal plant *Echinacea pallida* [32]. The loss of one of the two transgenes within the same T-DNA has been clearly demonstrated in transgenic wheat [33] and rice [34]. A single T-DNA insertion, with one copy of *nptII* and the foreign expression cassette (35S promoter/OD or 35S promoter/FAA), was found in transgenic plants OD5, OD14, FAA7, FAA10, and FAA14 (Figure 5). In addition, no introns are found in our *BPFAA* and *BPOD* genes (Supplementary Figure S3). In *Brassica napus*, no or two introns had been reported in *FAD2* genes (equivalent to our *BPOD* gene in this study) [35]. In *Medicago truncatula*, three *FAD2* genes have been reported; among them, *MtFAD2.1* had no intron, while *MtFAD2.2* had one intron and *MtFAD2.3* had two introns [20]. The exon–intron organization in *FAA* genes in other plant species has not been reported yet.

In this study, overexpression of *FAA* or *OD* did not significantly increase the mRNA expression of those genes, as revealed by qRT-PCR analysis (Figure 7). One possible explanation is the presence of multiple copies of the endogenous *FAA* or *OD* genes in the genome of WT *B. pilosa* (Figure 6); thus, overexpression of one homologous gene may not affect the total gene expression among those homologous genes. Nevertheless, preliminary HPLC profiling suggested that the seven polyacetylene (PA) compounds identified in transformants *OD*2, *OD*7, *OD*11, *OD*23, *FAA*10, *FAA*14, and *FAA*18 were slightly higher than in WT (Figure 9 and Figure 10).

The *fatty acid desaturase 2* (*FAD2*) gene encodes an enzyme that catalyzes the desaturation of oleic acid (C18:1) to linoleic acid (C18:2). Oleic acid and linoleic acid are two of the most abundant polyunsaturated fatty acids in plants [17]. For example, in safflower seed oil, oleic acid and linoleic acid together account for about 90% of the total fatty acids [14]. However, higher oleic acid content in oilseed crops is maintained by breeding programs because of the thermal stability of the resulting oil and its suitability as an edible oil [36]. Furthermore, oils containing higher oleic acid are beneficial for lowering cholesterol and reducing blood pressure [37]. Thus, by using the RNA interference (RNAi) technique to suppress the *FAD2* gene, higher expression of oleic acid and lower expression of linoleic acid have been reported in several plants, such as *Arabidopsis*, rice, cotton, and flax [38]. More recently, clustered regularly interspaced short palindromic repeats (CRISPR) and CRISPR-associated protein (Cas9) (CRISPR/Cas9) strategies have been developed for mutating the *FAD2* genes in oilseed crops. For example, in rapeseed (*Brassica napus*), two guide RNAs were designed for *BnaA.FAD2.a* (*FAD2_Aa*), which is one of four *FAD2* genes, followed by CRISPR/Cas9-mediated genome editing. Finally, two mature plants that contained the mutant alleles were obtained. Fatty acid composition analysis of seeds from homozygous lines revealed a statistically significant increase in oleic acid as compared with WT seeds [36]. Interestingly, the CRISPR/Cas9 system was successfully used to mutate all four copies of *BnaFAD2* in tetraploid rapeseed (*B. napus*), and the oleic acid content in the seeds of mutants was significantly increased compared with WT seeds [39]. In parallel, the production of high oleic acid and low linoleic acid due to the disruption of specific *FAD2* genes using CRISPR/Cas9-mediated mutagenesis was recently reported in rice [38], peanut [37], soybean [40], and tobacco [41]. It will be interesting to investigate whether the contents of oleic acid and the seven polyacetylenic compounds are affected if our *BPOD* gene (87% nucleotide sequence and 93% protein sequence identities to the sunflower *FAD2-2*) is disrupted by RNAi or CRISPR/Cas9 techniques.

In conclusion, herein, two full-length cDNAs that encode Δ12-fatty acid acetylenase (BPFAA) and Δ12-oleate desaturase (BPOD), which have been reported to be potentially involved in the polyacetylene biosynthesis pathway in other plants, were cloned from the medicinal herb *B. pilosa* var. *radiata*. These two cDNAs were constructed into plant expression vectors. Then, cotyledons were excised from *B. pilosa* plantlets that were grown in vitro, and *A. tumefaciens* infection, subculture, callus and shoot induction, and plant regeneration were conducted. Selection was conducted in an induction medium supplemented with 200 mg L^−1^ timentin to inhibit *Agrobacterium* growth and 200 mg L^−1^ kanamycin for selection. Finally, over 10 putative transgenic lines from each construct were obtained and grown in a greenhouse. A unique band of 795 bp (*nptII*, which confers kanamycin resistance) was detected in all the transgenic lines that we examined, but not in the WT, when *nptII*-specific primers were employed in genomic PCR analysis. This suggests that our transformation protocol for *B. pilosa* var. *radiata* was successful. The molecular characterization of these transformants was carried out.

## 4. Materials and Methods

### 4.1. Plant Material and Culture Conditions

Seeds of *Bidens pilosa* var. *radiata* were sterilized as previously described [30] and then germinated on MS basal medium (MS salts; 2% sucrose; 0.8% Bacto-agar; pH 5.7) [42] in a 25 °C growth chamber under a cycle of 16 h illumination (100 μmol m^−2^ s^−1^) and 8 h darkness. Explants of in vitro-grown plantlets were excised for *Agrobacterium*-mediated transformation.

### 4.2. Cloning of Full-Length cDNAs

To clone Δ12-fatty acid acetylenase and Δ12-oleate desaturase cDNAs in *B. pilosa* var. *radiata*, degenerate primers were designed based on homologous sequences in the National Center for Biotechnology Information (NCBI). Total RNA of *B. pilosa* var. *radiata* was used as a template to synthesize first-strand cDNA by 5′ and 3′ RACE using a SMARTer RACE 5′/3′ Kit (Clontech Laboratories is now Takara Bio USA, Mountain View, CA, USA) according to the manufacturer’s instructions. Specific primers for our FAA clone were designed according to Δ12 fatty acid acetylenase from sunflower (*Helianthus annuus*) (GenBank accession no. AY166773) [28], whereas specific primers for our OD clone were designed according to Δ12 oleate desaturase (FAD2-2) from sunflower (*H. annuus*) (GenBank accession no. AF251843) [19]. Oligonucleotide sequences of specific FAA primers (FAA-F, FAA-R, cFAA-f1, cFAA-f2, cFAA-r1, cFAA-r2) and specific OD primers (OD-F, OD-R, cOD-f1, cOD-f2, cOD-r1, cOD-r2) are listed in Supplementary Table S1. Protocols for gel purification, cloning into pGEM-T Easy Vector (Promega, Madison, WI, USA), and transformation into *Escherichia coli* JM109 competent cells were previously described [32]. Plasmid DNA was isolated and then sequenced. The nucleotide sequence that encodes Δ12-oleate desaturase from *B. pilosa* var. *radiata* was designated BPOD (1,152 bp) and submitted to NCBI with accession number MF318524, and the nucleotide sequence that encodes Δ12-fatty acid acetylenase from *B. pilosa* var. *radiata* was designated *BPFAA* (1134 bp) and submitted to NCBI with accession number MF318525.

### 4.3. Phylogenetic Analysis

Complete coding DNA sequences (CDSs) of the BPOD and BPFAA genes were studied with other oleate desaturase and fatty acid acetylenase complete CDSs obtained from the GenBank database. Desaturase and acetylenase CDSs were analyzed, respectively, and aligned using Clustal W [43]. Phylogenetic studies were carried out using the maximum likelihood method based on the Tamura–Nei model [29] in the MEGA7 software [44]. Phylogenetic analysis of these CDSs is represented by a bootstrap consensus tree (1000 replicates) in a traditional branch style.

### 4.4. Construction of Expression Vectors and Agrobacterium-mediated Transformation

Two full-length cDNAs that encode BPFAA (1134 bp) and BPOD (1152 bp) were separately cloned into the Gateway expression vector pK2GW7 [carrying neomycin phosphotransferase II (*nptII*) for kanamycin resistance], resulting in pBPFAA and pBPOD, respectively. These two vectors (i.e., pBPFAA and pBPOD) were transformed separately into *Agrobacterium tumefaciens* strain LBA4404 by electroporation (Bio-Rad Gene Pulser II, Hercules, CA, USA), and the individual *Agrobacterium* culture carrying a single transformation vector was used for plant transformation as previously described [30]. Briefly, cotyledon explants were excised from *B. pilosa* plantlets grown in vitro. *Agrobacterium*-mediated transformation, subculture, callus induction, and plant regeneration were performed. Selection was conducted in a medium containing MS salts, 2% sucrose, 1 mg L^−1^ BA, 0.5 mg L^−1^ IAA, and 0.8% Bacto-agar supplemented with 200 mg L^−1^ timentin and 200 mg L^−1^ kanamycin.

### 4.5. Transgenic Plant Verification by Genomic PCR Analysis

Total genomic DNA was extracted from the green leaves of wild-type (WT) and putative transgenic plants using the CTAB method [45]. PCR was carried out with the following primer sets (Supplementary Table S1): 35S Pro-F1 and OD-R for amplification of a 1366-bp-long DNA fragment corresponding to the partial CaMV 35S promoter and partial *OD* sequences; 35S Pro-F1 and FAA-r2 for amplification of the 764-bp-long DNA fragment corresponding to the partial CaMV 35S promoter and partial *FAA* sequences; Kan-F and Kan-R for amplification of the 795-bp-long corresponding to the full-length *nptII* gene for kanamycin resistance [46]. Conditions for PCR and gel electrophoresis were as previously described [30].

### 4.6. Southern Blot Analysis

For Southern blot analysis, 20 μg of genomic DNA from putative transgenic and WT plants was independently digested with *Eco*RI. Probe preparation and detection of the non-isotope Digoxigenin (DIG)-labeled PCR product for the kanamycin selection marker *nptII* were carried out as previously described [30].

To determine the endogenous copy numbers of the OD and FAA genes, 20 μg of genomic DNA from WT plants was digested by various restriction enzymes for *Afl*II, *Eco*RI, *Nco*I, *Sac*I, and *Pac*I. No restriction cutting sites were found using these restriction enzymes in the full-length cDNAs of either FAA or OD. For gene-specific probes, primers FAA-F and FAA-R (Supplementary Table S1) were used to amplify the 1134-bp-long DNA fragment corresponding to the full-length cDNA of FAA, and primers OD-F and OD-R (Supplementary Table S1) were used to amplify the 1152-bp-long DNA fragment corresponding to the full-length cDNA of the OD gene. The protocols for PCR amplification, gel purification, blotting onto a nylon membrane, preparing the non-isotope DIG-labeled PCR probe, hybridization, and detection were as previously described [30].

### 4.7. Quantitative Real-Time PCR Analysis

Total RNA was isolated from the green leaves of WT and transgenic plants by CTAB [47]. Quantitative real-time PCR (qRT-PCR) analysis was carried out as previously described [32]. Specific primer sets for the amplification of DNA fragments for FAA (187 bp), *OD* (234 bp), and ribosomal protein L2 (100 bp; internal control) from *B. pilosa* are listed in Supplementary Table S1. Three biological replicates were used for quantification.

### 4.8. Western Blot Analysis

One hundred milligrams of leaf tissue from WT and transgenic plants was harvested and frozen immediately in liquid nitrogen; the tissue was ground into a fine powder, and 400 μL of protein extraction buffer (50 mm Tris-HCL, pH 7.0; 0.5 mm EDTA; 1× protease inhibitor) was added in a 2 mL micro-centrifuge tube. The tube was vortexed for 10 s, put on ice for 20 min, and then centrifuged at 16,000 rpm for 30 min at 4 °C. The supernatant was transferred into a fresh micro-centrifuge tube, and protein concentration was determined by DC Protein Assay Reagent (Bio-Rad) with bovine serum albumin as the standard. To produce the antibody against *BPOD*, oligopeptide (N’-SHR RHH SNT GSI EHD EVF-C’) conjugated to the carrier protein OVA was synthesized, HPLC purified, and then used as an antigen to immunize rabbits. Immunization and serum collection were performed by LTK BioLaboratories (New Taipei City, Taiwan). For immunoblot analysis, 30 μg of total protein was electrophoretically separated on 10% SDS-polyacrylamide gel and then transferred onto a PVDF membrane. The blot was incubated with anti-*OD* antibody or anti-actin antibody (control) and then visualized by incubation with horseradish phosphatase (HRP)-conjugated goat anti-rabbit IgG, followed by chemiluminescent HRP substrate detection. To ensure equal loading of protein, the SDS-polyacrylamide gel was stained with Coomassie Brilliant Blue.

### 4.9. High-Performance Liquid Chromatography Analysis

HPLC analysis was carried out as described previously [4,6] with modifications. Briefly, one-month-old leaves (100 mg) from WT and selected transgenic plants were harvested and frozen immediately in liquid nitrogen; the tissues were ground into a fine powder and transferred into a micro-centrifuge tube. Then, 1 mL of cold 70% ethanol was added into each tube and vortexed for 10 s. After sonication for 20 min (samples were kept in an ice-water bath), tubes were centrifuged for 10 min at 12,000× *g*. Supernatant from each sample was filtered out by using a 1-mL syringe and 0.2 μm PTFE membrane into a fresh micro-centrifuge tube. HPLC analysis was conducted using an Agilent 1200 Chemstation HPLC system and a C18 reverse-phase column (Phenomenex Luna 5 μ C18, 250 mm × 4.6 mm, Torrance, CA, USA). Polyacetylenic compounds were monitored at 245 nm. The mobile phase consisted of H_2_O (A) and methanol (B), and separations were performed using the following gradients: 60% B from 0 to 10 min, 70% B from 10 to 15 min, 80% B from 15 to 30 min, 90% B 30 to 40 min, 100% B 40 to 45 min, 100% B 45 to 65 min, and 60% B from 65 to 85 min. The injection volume was 30 μL.

Polyacetylenic compounds, including 2-β-D-glucopyranosyloxy-1-hydroxy-5(*E*)-tridecene-7,9,11-triyne (compound **1**), 3-β-D-glucopyranosyloxy-1-hydroxy-6(*E*)-tridecene-tetradecene-8,10,12-triyne (compound **2**), 2-β-D-glucopyranosyloxy-1-hydroxy-trideca-5,7,9,11-tetrayne (also known as cytopiloyne, compound **3**), 1,2-dihydroxy-5(*E*)-tridecene-7,9,11-triyne (compound **4**), 1,3-dihydroxy-6(*E*)-tetradecene-8,10,12-triyne (compound **5**), 1,2-dihydroxy-trideca-5,7,9,11-tetrayne (compound **6**), and 1-phenylhepta-1,3,5-triyne (compound **7**), were isolated from *B. pilosa* var. *radiata* extract in this study and have been determined by NMR spectroscopy, as published elsewhere [4,8,48].

## Supplementary Materials

The following is available online at https://www.mdpi.com/2223-7747/9/11/1483/s1: Figure S1: Construction of expression vectors for plant transformation; Figure S2: Typical photos demonstrating shoot regeneration under selection medium and then plant regeneration of transgenic *Bidens pilosa* plants by transforming two expression vectors and *Agrobacterium*-mediated method; Figure S3: Examination of PCR products between genomic DNA and cDNA for *FAA* and *OD* genes in *Bidens pilosa*; Figure S4: Representative HPLC profiles of WT and a few randomly selected transformants; Table S1: Primers used for this study.

## Abbreviations

*BA*	6-Benzyladenine
BPFAA	Δ12-Fatty acid acetylenase from *Bidens pilosa* var. *radiata*
BPOD	Δ12-Oleate desaturase from *Bidens pilosa* var. *radiata*
CaMV	Cauliflower mosaic virus
*FAD2*	*Fatty acid desaturase 2*
HPLC	High-performance liquid chromatography
*hptII*	Hygromycin phosphotransferase II
IAA	Indole-3-acetic acid
*nptII*	Neomycin phosphotransferase II
qRT-PCR	Quantitative real time-PCR
WT	Wild-type
