# Supplementary material for: Transformation and Characterization of Δ12-Fatty Acid Acetylenase and Δ12-Oleate Desaturase Potentially Involved in the Polyacetylene Biosynthetic Pathway from Bidens pilosa
Source: Plants (Basel). 2020 Nov 3;9(11):1483. doi: 10.3390/plants9111483 (PMC7693981; doi:10.3390/plants9111483)
Supplement: Supplementary file 1 [file plants-09-01483-s001.pdf]

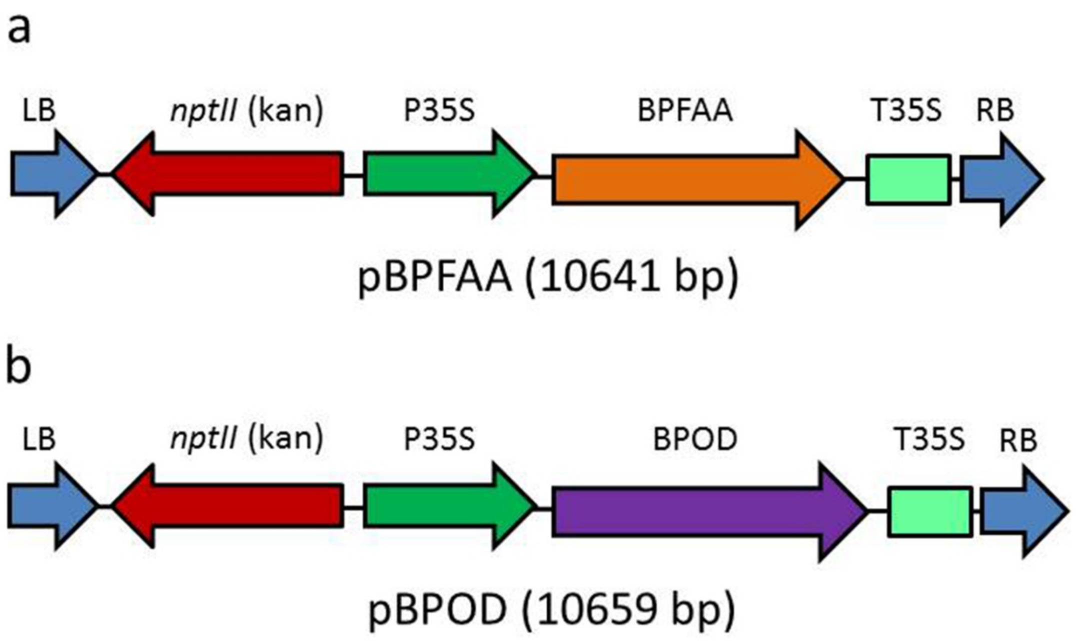

Supplementary Figure S1. Construction of expression vectors for plant transformation. (**a**) pBPFAA. (**b**) pBPOD.

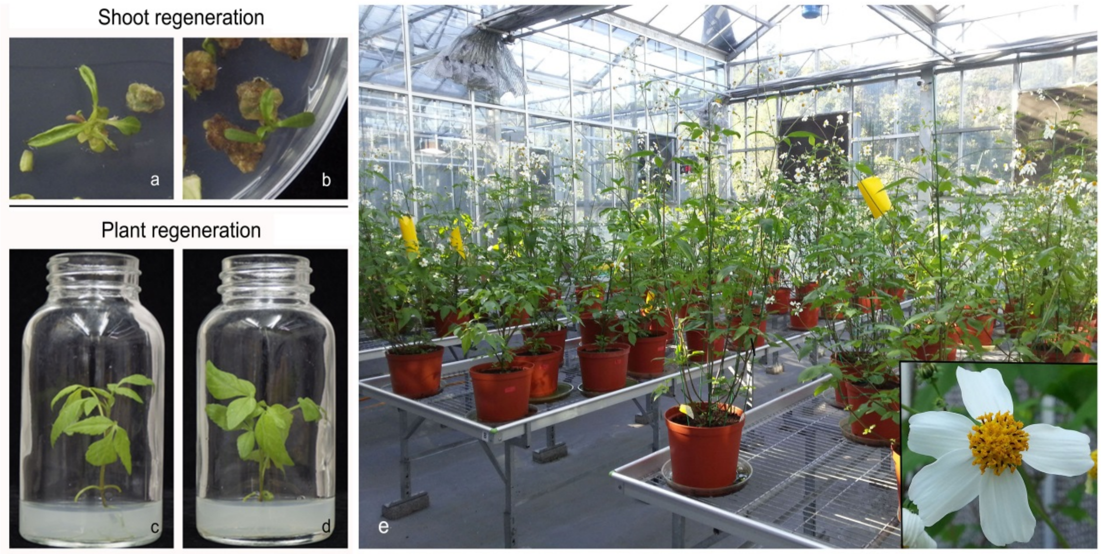

Supplementary Figure S2. Typical photos demonstrating shoot regeneration under selection medium and then plant regeneration of transgenic *Bidens pilosa* plants by transforming two expression vectors and *Agrobacterium*-mediated method. Panels (a) and (c): transgenic FAA lines; panels (b) and (d): transgenic OD lines; panel (e): transgenic plants together with wild-type plants were grown in greenhouse. Mature flower from a transgenic plant is shown in a small panel at (e).

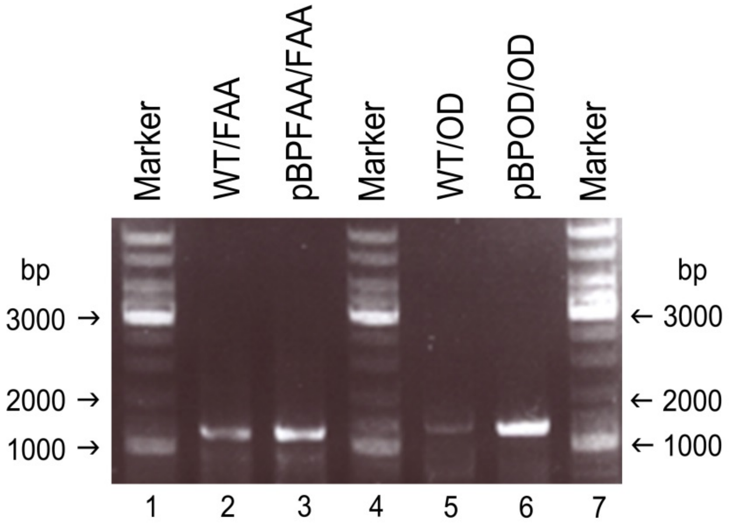

Supplementary Figure S3. Examination of PCR products between genomic DNA and cDNA for *FAA* and *OD* genes in *Bidens pilosa*. Plasmids pBPFAA and pBPOD were used as cDNA template, PCR was carried out using specific FAA (FAA-F and FAA-R) and OD (OD-F and OD-R) primers to amplify full-length regions of FAA (1134 bp) and OD (1152 bp), and compared with PCR products of genomic DNA isolated from wild-type *B. pilosa* var. *radiata*.

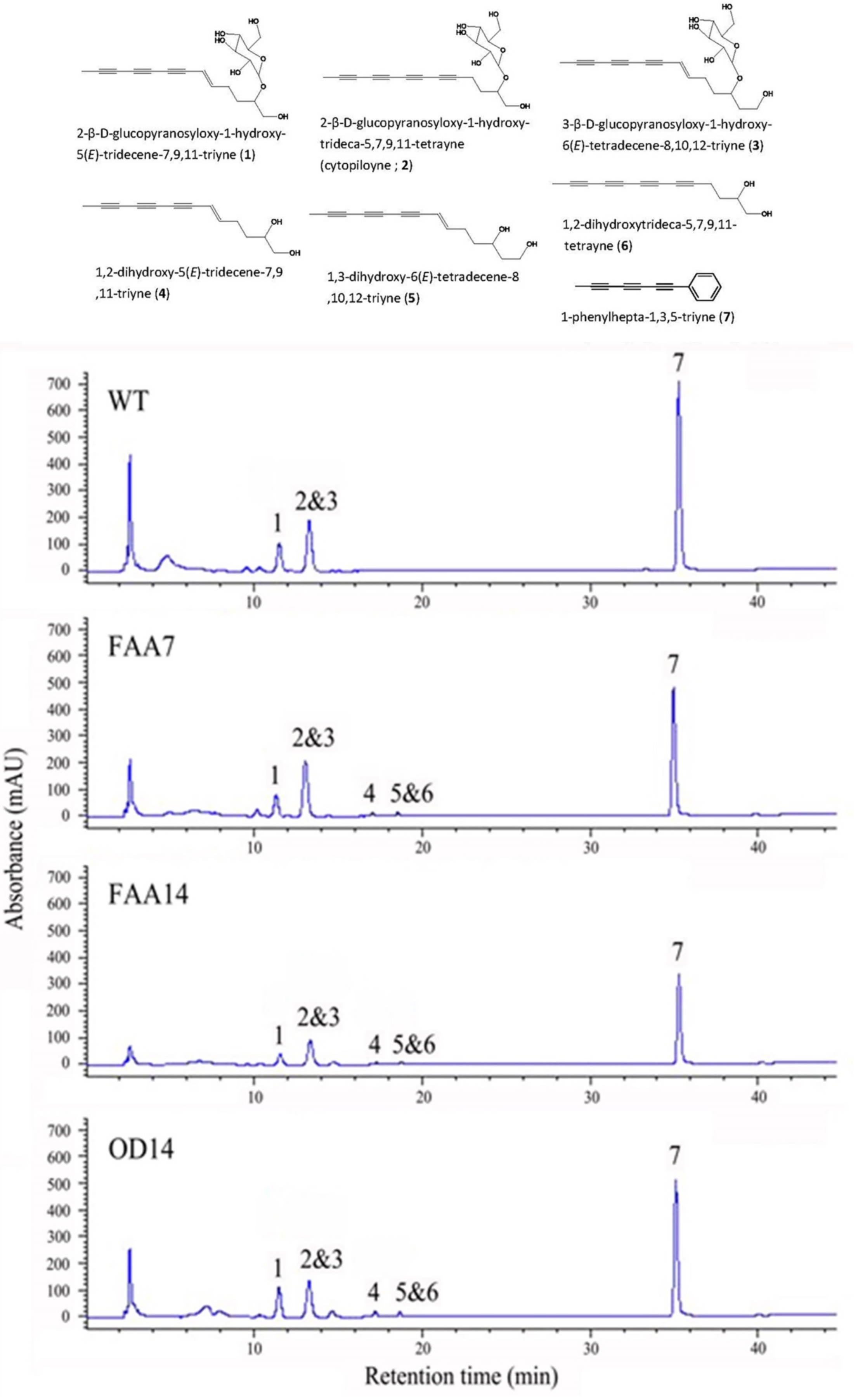
2-β-D-glucopyranosyloxy-1-hydroxy-5(E)-tridecene-7,9,11-triyne (1)
2-β-D-glucopyranosyloxy-1-hydroxy-trideca-5,7,9,11-tetrayne (cytopiloyne ; 2)
3-β-D-glucopyranosyloxy-1-hydroxy-6(E)-tetradecene-8,10,12-triyne (3)
1,2-dihydroxy-5(E)-tridecene-7,9,11-triyne (4)
1,3-dihydroxy-6(E)-tetradecene-8,10,12-triyne (5)
1,2-dihydroxytrideca-5,7,9,11-tetrayne (6)
1-phenylhepta-1,3,5-triyne (7)
WT
FAA7
FAA14
OD14
1
2&3
4
5&6
7
Absorbance (mAU)
Retention time (min)

Supplementary Figure S4. Representative HPLC profiles of WT and a few randomly selected transformants. Chemical structures for seven polyacetylenic (PA) compounds have been determined by NMR spectroscopy as previous report [8]. The UV detection wave length was set at 245 nm. Retention times for PA compounds **1** (11 min), **2&3** (co-eluted at 13 min), **4** (17 min), **5&6** (co-eluted at 18 min) and **7** (35 min) were determined as previously described [4,8,48].

Supplementary Table S1. Primers used for this study.

Primer	Sequence (5'→3')
35S Pro-F1	TGA TAT CTC CAC TGA CGT
FAA-r2	CAA AGT GAA CAC TCG AC
FAA-F	ATG GGT GCA GGT GGC CGG
FAA-R	TTA AAA CTT ATG GTA CCA
OD-F	ATG GGT GCA GGC GGG CGA
OD-R	TCA TAT GTT ATT ACG GTA CCA A
Kan-F	ATG ATT GAA CAA GAT GGA
Kan-R	TCA GAA GAA CTC GTC AAG
qFAA-F2	GCA TGC TCG GTG TGC TTT AC
qFAA-R2	GCC CCT TTG ATC CAG TTC CA
qOD-F3	AAC TCT AAC CCT TGG CTG GC
qOD-R3	AAC CCA TTC ACC ACG AGC AA
qL2-F	CAT CAT GTG GTA AAG GTC GTA ATG
qL2-R	CGC TTA TGA CCT CCC CCT CTA
cOD-f1	GGT CAT AGC CCA CGA GTG CGG
cOD-f2	CTA TGA CCG CTT CGC ATG CCA
cOD-r1	CCT GTG TTC GAG TGG TGG CG
cOD-r2	GGC ATT GTT GAG AAC AGA TGG TG
cFAA-f1	GAA TGC GGT CAC CAC GCC TAT
cFAA-f2	AAC CAC TTC GAT CCA TTA AG
cFAA-r1	AGA GAG ATG GGT GTG GTG ATT
cFAA-r2	CCA AAG TGA ACA CTC GAC CAG
